# The Rationale for Combining Normothermic Liver Machine Perfusion with Continuous Renal Replacement Therapy to Maintain Physiological Perfusate during Ex Vivo Organ Perfusion

**DOI:** 10.3390/jcm13175214

**Published:** 2024-09-03

**Authors:** Federico Nalesso, Alessandra Bertacco, Elisabetta Bettin, Martina Cacciapuoti, Marco Bogo, Leda Cattarin, Jacopo Lanari, Alessandro Furlanetto, Alessia Lanubile, Enrico Gringeri, Lorenzo A. Calò, Umberto Cillo

**Affiliations:** 1 Nephrology, Department of Medicine (DIMED), University of Padua, 35128 Padua, Italy; elisabetta.bettin@aopd.veneto.it (E.B.); leda.cattarin@aopd.veneto.it (L.C.);; 2 Hepato-Biliary-Pancreatic and Liver Transplant Unit “Chirurgia Generale 2”, Padua University Hospital, 35128 Padova, Italyjacopo.lanari@unipd.it (J.L.); cillo@unipd.it (U.C.); 3 Pharmaceutical Science Department (DSF), University of Padua, 35131 Padua, Italy

**Keywords:** continuous kidney replacement therapy, liver normothermic perfusion ex vivo, normothermic machine perfusion, organ perfusion ex vivo, organ transplant

## Abstract

**Background:** The possibility of keeping liver grafts viable and functioning until transplantation has been explored since the 1950s. However, the current modalities of Normothermic Machine Perfusion (NMP) have shown several limitations, such as the inability to correct electrolytes and pH derangements efficiently. Combining NMP with continuous kidney replacement therapy (CKRT) might provide a promising new model to overcome these issues. **Methods:** An NMP that covers the organ perfusion, oxygenation, carbon dioxide removal, and thermal balance was connected to a CKRT circuit to ensure physiological hydro-electrolytes, acid–base balance, and catabolite removal from the perfusate. **Results:** The integration of NMP and CKRT maintains a neoplastic liver in a perfusion system with physiological perfusate for 100 h. CKRT re-established and maintained the hydro-electrolyte and acid–base status throughout the 100 h of perfusion. Significant limitations were the need for frequent monitoring of electrolytes and acid–base disorders and the loss of low molecular weight nutrients, which have to be replenished by manual infusion into the system. **Conclusions:** This novel CKRT-NMP integrated system may represent a practical and versatile model to support organs’ perfusion and extend preservation times. Further experiments are needed to fix monitoring and adjusting processes.

## 1. Introduction

Liver transplantation has become increasingly influential in treating various diseases and improving patient survival, leading to higher demand. Advances in preoperative care, surgical techniques, and postoperative management have made it possible to transplant high-risk patients successfully. Hypothermic machine perfusion (HMP) and normothermic machine perfusion (NMP) [1] further enhance these advancements by safely using suboptimal grafts through objective viability assessments and minimizing cold ischemia. This technology offers physiological and extended preservation times, benefiting both grafts and surgical logistics, representing a significant advancement in transplantation practices [2,3,4,5].

Claude Bernard described in the 1950s the first model of ex vivo liver perfusion. In 1935, Lindbergh-Carrel designed the first perfusion pump to provide an ex vivo perfusion for the thyroid gland [6]. The limited technology in this new field of organ perfusion was unsuccessful in maintaining organ function, and organ transplantation preservation was based on the principles of static cold storage (SCS). In 1967, Brettschneider et al. described the first animal model of ex situ machine preservation for liver transplantation; after that, it was applied by Starzl to the first human liver transplantation [7]. The lack of technology and the initial setbacks in organ perfusion had limited the development of organ perfusion machines by driving interest in the clinical use of cold storage solutions until 2013, when the world’s first liver transplant was performed by Friend and colleagues in England. The NMP beneficial effect provided by the first randomized controlled trial (RCT) on NMP in 2018 [8] represented the introduction of the clinical practice of normothermic perfusion [9] following the previous hypothermic perfusion studies performed by Guarrera et al. [10]. 

The typical concept of NMP is maintaining organs ex vivo at physiological temperatures while maintaining their metabolic functions and preserving their function for the transplant. The preserved aerobic metabolism would reduce the incidence of ischemia-reperfusion injury (IRI) at the moment of transplantation in the recipient. To mimic the physiologic condition of organ perfusion, the NMP has to pump perfusate through the portal vein and hepatic artery at different pressures while maintaining the temperature at 37 °C. 

In recent years, the clinical use of NMP has increased, and there is growing evidence of its beneficial effect on liver transplantation [11]. This is evident and useful in the expanded criteria donor (ECD) setting [11,12]. NMP may also become an optimal platform for regenerative and therapeutic medicine purposes [13,14,15].

Three commercially available normothermic perfusion devices have been used in clinical trials: OrganOx Metra, TransMedics Organ Care System, and XVIVO perfusion. Similar principles have been used in all of them. The differences are in portability, automation, recirculating perfusate pressure and perfusate flow pulsatility, substrate type and delivery, and perfusion through the portal vein and hepatic artery. The lack of a complex purification system to maintain physiological properties at a biochemical and chemical level is a common feature of this technology. This limitation of current NMP systems lies in their inability to continuously correct the perfusate composition. From a rudimentary and classical point of view, these systems can be associated with intermittent purification systems (intermittent hemodialysis), which rely solely on executing an intermittent diffusive process, resulting in a rapid and only transitory correction of the various modifiable parameters. In the case of a rudimental continuous purification system integrated into the NMP, diffusion is the only physical principle used in the perfuse purification technique without a safe and easy-to-use system for fluid removal. The major limitation of the NMP technology lies in its inability to continuously correct electrolyte concentrations and pH and maintain the fluid balance during drug and fluid administration, which determines hemoglobin dilution over time and catabolite accumulation. In fact, these issues can also lead to increased perfusate sodium concentration due to sodium bicarbonate administration for pH maintenance and sodium-containing drug administration. At the same time, potassium levels rise due to hemolysis due to mechanical pumps and from intracellular release by the perfused organ. Calcium, magnesium, and phosphate levels can fluctuate due to drug administration, cell metabolism, and pH-dependent protein binding, and these variations can lead to significant metabolic organ changes, especially in cases of hypocalcemia and hypophosphatemia [16,17]. At the time of NMP priming, the perfusate exhibits non-physiological electrolyte concentrations and pH due to its intrinsic artificial composition, which involves mixing different solutions depending on the various protocols used in NMP. For instance, electrolyte changes are expected due to the regular potassium release from the perfusate-damaged red blood cells and the sodium load required to administer sodium bicarbonate to ensure physiological perfusate pH. Upon initiating organ perfusion, the potassium released into the perfusate by the injured cells and the relative metabolic acidosis result in further release of intracellular potassium, worsening its levels in the perfusate. The NMP priming perfusate and its electrolyte concentration and acid–base state are not physiological, even in the initial phases of organ perfusion. This is due to the initial perfusate composition and the first organ perfusion that subsequently releases a series of catabolites and ions into the bloodstream due to the tissue damage from the previous surgical ischemia phase. During the perfusion, the cells metabolic activity results in the continuous production of catabolites and consumption of bicarbonates, causing metabolic acidosis and progressive “intoxication” of the perfusate that usually presents a close system with the total volume ranging between 1.5 and 3 L. The presence of a non-physiological perfusate has the potential to induce alterations in cellular metabolism. To maintain optimal cellular function, it is essential to ensure the continuous removal of catabolites and the gain of molecules used by metabolism, such as glucose, phosphate, and various nutrients (vitamins and trace elements). 

Furthermore, it is crucial to provide an adequate oxygen supply and remove carbon dioxide while maintaining the partial pressures of these gases within physiological ranges. It is challenging to control the perfusate pH as it is necessary to simultaneously guarantee correct pO_2_ and pCO_2_ levels in the presence of bicarbonates that maintain the pH within a physiological range (7.35–7.45) [18,19]. Based on the organ’s metabolic activity, this process requires the continuous modulation of the airflow for the removal of CO_2_ (mL/min) and the administration of sodium bicarbonate to ensure the maintenance of adequate bicarbonate levels. The progressive sodium gain within a closed system finally increases its perfusate concentration, determining a significant rise in osmolarity and the exit of water from the cells. This phenomenon is further enhanced by cellular metabolism catabolites accumulated in the extracellular space and the perfusate. 

The NMP limitations necessitate the continuous monitoring of ionic levels and acid–base status, with manual interventions to maintain a physiological environment for the perfused organ by infusing deficient molecules and attempting to remove those in excess. From this perspective, the potential for combining organ perfusion with an integrated system capable of eliminating excess molecules and supplying defective molecules could facilitate the implementation of a new NMP system that ensures the achievement and maintenance of a biochemically and chemically physiological state despite the possibility of alterations in cellular metabolism and perfusion complications such as lysis of red blood cells and organ cell necrosis. The extracorporeal blood purification currently used in the continuous modality (Continuous Kidney Replacement Therapy, CKRT [20,21]) in patients suffering from Acute Kidney Injury (AKI) [22,23] could potentially satisfy all the purification needs and NMP requests by providing a versatile and customizable tool to meet metabolic needs during organ perfusion, which could be conducted for prolonged periods. The ability to maintain a chemically and biochemically physiological perfusate such as the blood in the human body may provide the first step towards designing and implementing an NMP system that can guarantee the next future ex vivo perfusion of organs for longer periods of time compared to the existing technology.

Recently, introducing a new combined NMP and CKRT system has made it possible to obtain normothermic perfusion of a human tumoral liver for 17 days, opening new perspectives for organ perfusion over long periods [24] thanks to the preliminary combination of NMP with CKRT. 

Combining ex vivo organ perfusion with extracorporeal perfusate purification requires specific hardware provided by specific NMP and CKRT monitors. The current market offers a range of organ perfusion and extracorporeal blood purification technologies, typically provided by different modules that are impossible to combine in a single perfusion machine. To date, no available hardware has been developed that can combine both processes simultaneously in a simple way with a friendly interface to treatment management over time. Consequently, to combine these two processes, it is necessary to integrate different technologies manually.

**Figure 1:**
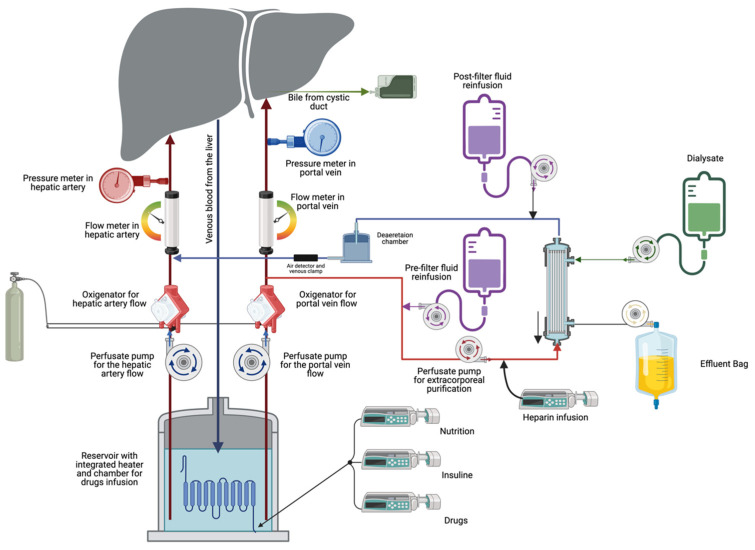
NMP combined with CKRT for the liver perfusion.

The combination of NMP with CKRT involves the connection of the two perfusate circuits so that the NMP guarantees organ perfusion, oxygenation, carbon dioxide removal, and thermal balance. At the same time, the CKRT maintains a physiological hydro-electrolyte and acid–base balance and provides catabolite removal and the gain of deficient substances into the perfusate. Figure 1 shows the ideal NMP simultaneously performing the heart, lung, and kidney function during liver perfusion. The continuous nature of CKRT, in contrast to the intermittent approach of classical hemodialysis (HD), enables the maintenance of a constant perfusate molecule level. This is achieved by preventing fluctuations in catabolite concentrations, ions, and acid–base balance while ensuring a stable fluid balance.

Typically, the CKRT monitor is connected to the NMP by two specific lines. The arterial line (A) is designated to collect the perfusate to be purified, while the venous line (V) is designed to return the perfusate to the NMP circuit. In the connection design, it is essential to ensure that the arterial and venous lines are suitably connected so as not to disrupt the organ perfusion flow generated by the NMP pump(s) or induce alterations in the pressures in the CKRT circuit, as this could impede the purification treatment over the perfusion duration. 

Moreover, the line A and V connection ports have to be designed to avoid technical problems, ensuring the effectiveness of the purification process and the perfusion flow. Figure 1 shows one of the ideal configurations of the connections between the two NMP and CKRT monitors. In this way, any change in perfusate flow in the purification circuit is automatically compensated by feedback from the monitor NMP.

The NMP system ensures organ perfusion by regulating the perfusate flow through feedback systems based on perfusion resistances and pressures, perfusate oxygenation, and carbon dioxide removal. It is of paramount importance that the flow of perfusate removed or reinfused into the circuit does not influence flow meters or pressure switches, thereby altering the perfusion flow estimated according to the feedback operating in the NMP system.

In this combined configuration, the CKRT monitor ensures continuous perfusate purification through diffusive (hemodialysis, HD) and convective (hemofiltration, HF) processes, which are appropriately combined in the hemodiafiltration technique (HDF) [20], as shown in Figure 1. This enables the achievement of an adequate purification dose (mL/min) and a suitable target of molecules to be removed [25], in the specific case of all catabolites from the organ metabolism or cell damage. The continuous characteristic of the purification in CKRT allows optimal levels of molecules in the perfusate to be achieved and maintained over time, which cannot be achieved with an intermittent process such as that used in the past in some NMP technologies. In CKRT, in particular, diffusion allows for removing excess electrolytes from the perfusate while ensuring the back diffusion of deficient electrolytes or valuable substances, such as glucose and bicarbonate. The convective process is more effective than the diffusive process in removing catabolites with a higher molecular weight (beyond the weight of medium molecules). The most helpful method to achieve the most significant removal of catabolites and to efficiently correct electrolyte and acid–base imbalances is post-dilution hemodiafiltration (HDF-post dilution), which does not require any pre-dilution in the filter. This process necessitates post-dilution reinfusion only, which can facilitate the infusion of replenishing substances, such as glucose, phosphate, and bicarbonate, and other substances directly into the venous line of the perfusate balancing and avoiding the manual infusions in the NMP circuit normally operated by the use of more than one solution by syringes. The absence of a pre-filter reinfusion does not result in a dilution of the perfusate, thereby ensuring that the diffusive and convective doses are as effective as possible in purifying the perfusate. The combination of diffusion and convection with post-dilution reinfusion in CKRT represents an innovative technology implementation that facilitates the most effective perfusate purification as processes employed in blood purification in renal failure patients.

The chemical characteristics of the hemodiafiltration fluids employed for the post-dilution convection and dialysis processes can be modified according to the commercial preparations available and the clinical needs requiring adjustment of the ion concentration in relation to the duration of treatment [26,27]. The correction of the ionic concentrations or the addition of electrolytes or other substances, such as glucose, according to the perfusion needs can be required to achieve the best hemodiafiltration fluid for perfusate purification. The use of specific membranes in the hemodiafiltration filter enables the spectrum of removable molecules to be broadened by varying the Molecular Weight Retention Onset (MWRO) and Molecular Weight Cut-off (MWCO) and employing surface absorption [28]. The dialyzer selection has to be based on the specific purification requirements and the type of treatment to be performed (HD, HF, or HDF) [20]. To ensure that the characteristics of the dialyzer satisfy the purification needs without causing complications such as the loss of albumin, it is essential to consider the factors above to confirm the match between the treatment and the filter used [29]. The potential for utilizing specific filters with elevated cut-offs or engineered membrane surfaces enables the further reduction of inflammatory molecules, such as cytokines, and the safeguarding of the organ from the risk of inflammatory damage.

In our study, we combined CKRT and NMP to reach the best physiological perfusate, aiming to create a preclinical platform for future ex vivo organ manipulation. In detail, we describe in this article a single CKRT-NMP exemplar of our protocol that we have already applied to eight cases of cancer liver NMP. The possibility of recovering and preserving the physiological characteristics of perfusate is the first step to maintaining a liver in homeostasis for prolonged periods, representing an experimental challenge with extremely relevant future implications. If confirmed feasible, it will open a wide span of ex vivo organ manipulation scenarios as gene therapy delivery and drug testing in metabolic (e.g., NASH) cirrhotic and neoplastic disease. Moreover, the liver regeneration strategy could be tested, considering the long perfusion time (more than 2 weeks) under physiological conditions. The objective of our study is to demonstrate that CKRT maintains a more stable and physiological perfusate and, consequently, the rationale to use CKRT during NMP could serve as the initial phase in developing this innovative platform. The results achieved showed excellent biochemical stability during the ex vivo perfusion, allowing for a prolongation of the normothermic perfusion, confirming the rational use of CKRT. The focus of our article is on the technical aspects related to the perfusate purification being a clinical outcome outside the scope of this study.

## 2. Materials and Methods

In this study, we used the Liver Assist Organ Perfusion System (Xvivo, Gronigen, The Netherlands) for liver perfusion and the Prismax system with ST-150 set (Baxter, Deerfield, IL, USA) for the perfusate purification. Depending on the purification requirements, the fluids used for the hemodiafiltration were Prismasol 4, Prismasol 2, and Phoxilium (Baxter, Deerfield, IL, USA). Haemogas analyses were performed at regular intervals over time to determine the composition of the most appropriate hemodiafiltration fluid and adjust the electrolyte content of the available fluids. Blood flow was set at 200 mL/min, dialysate flow at 1000 mL/h, and post-dilution reinfusion at 500 mL/h, with the pre-dilution reinfusion disabled to eliminate the predilution of the perfusate to be purified. The hourly weight loss was set according to the daily fluid balance requirements. After the blood pump, a continuous dose of 1000 IU/h of unfractionated sodium heparin was infused into line A of the CKRT to prevent extracorporeal circuit clotting. Perfusate temperature was maintained only by the NMP monitor heater with the TherMax disabled. The decision to disable the TherMax heater ensured that any potential bias introduced by the NMP heater was eliminated. Consequently, the heat balance and temperature of the perfusate were solely controlled by the NMP heater.

The liver used for perfusion (weight: 1100 g) came from patients with unresectable cholangiocarcinoma undergoing deceased donor transplantation according to the protocol approved by the Ethics Committee for the Azienda Ospedale-Università Padova (Comitato Etico per la Sperimentazione Clinica della provincia di Padova, protocol number AOP2678, approval document on 20 June 2022).

To monitor the perfusate composition, regular blood tests were performed over the liver perfusion to assess the impact of CKRT on ions and acid–base status. Liver viability was defined using the criteria proposed in the VITTAL and DHOPE-COR-NMP trials and are not included in this article that focuses on the role of perfusate purification by CKRT.

## 3. Results

**Figure 2:**
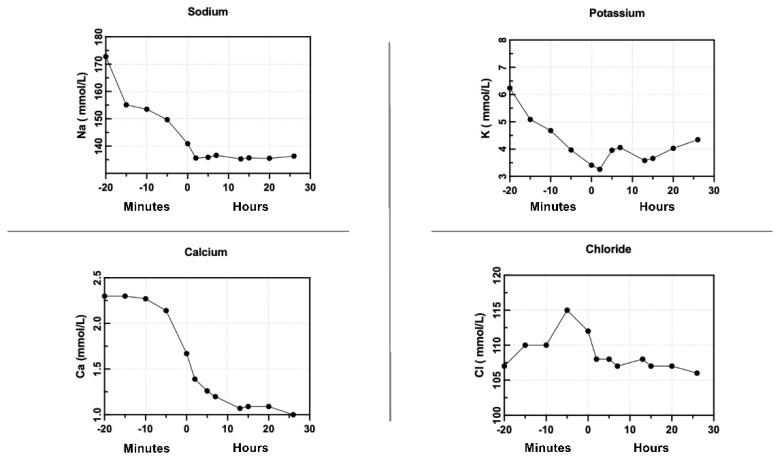
Electrolytes before and in the first 24 h of perfusion.

**Figure 3:**
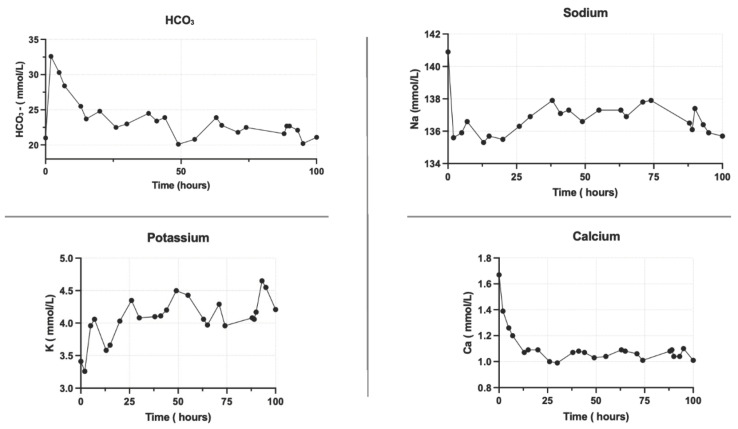
Bicarbonates and electrolytes for the first 100 h of liver perfusion.

**Figure 4:**
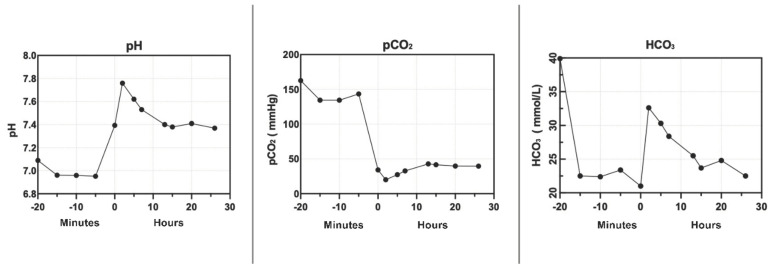
Basic acid status before and during the first 24 h of perfusion.

**Figure 5:**
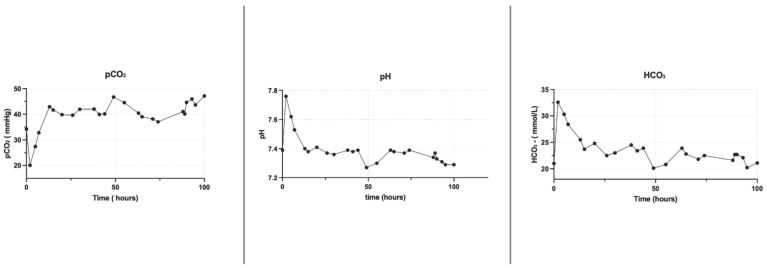
Basic acid status for the first 100 h of liver perfusion.

In our experiment, the combination of NMP and CKRT in liver perfusion allowed the initiation of organ perfusion with a physiological perfusate throughout the entire duration of the perfusion. The chemical composition of the perfusate before liver perfusion is subjected to a 20-minute purification process, which determines the achievement of a physiological perfusate at the time of organ perfusion and its maintenance for the entire duration of this study (Figure 2, Figure 3, Figure 4 and Figure 5).

### 3.1. Electrolyte Balance

Figure 2 shows the correction of the perfusate ions concentration before the beginning of liver perfusion and the maintenance of constant physiological ions concentration in the perfusate after the perfusion. Figure 3 demonstrates the stability of ions in the physiological range over 100 h of perfusion.

As shown in Figure 1, Figure 2 and Figure 3, the physiological ion concentrations were restored before organ perfusion and maintained for the duration of perfusion with the combination of NMP and CKRT.

### 3.2. Acid–Base Status

The use of NMP in combination with CKRT permitted the bicarbonate levels in the perfusate to be regulated in relation to the CO_2_ levels obtained with the oxygenator into the NMP circuit. This enabled the pH to be controlled before perfusion and its maintenance over time. The CKRT allows a source of bicarbonate to be continuously supplied to the system to buffer its reduction. Figure 4 illustrates the pH, pCO_2_, and bicarbonate levels before liver perfusion, and Figure 5 over the subsequent 100 h.

These results demonstrate that CKRT integrated into the NMP system allowed obtaining and maintaining a physiological perfusate during the 100-hour perfusion.

**Figure 6:**
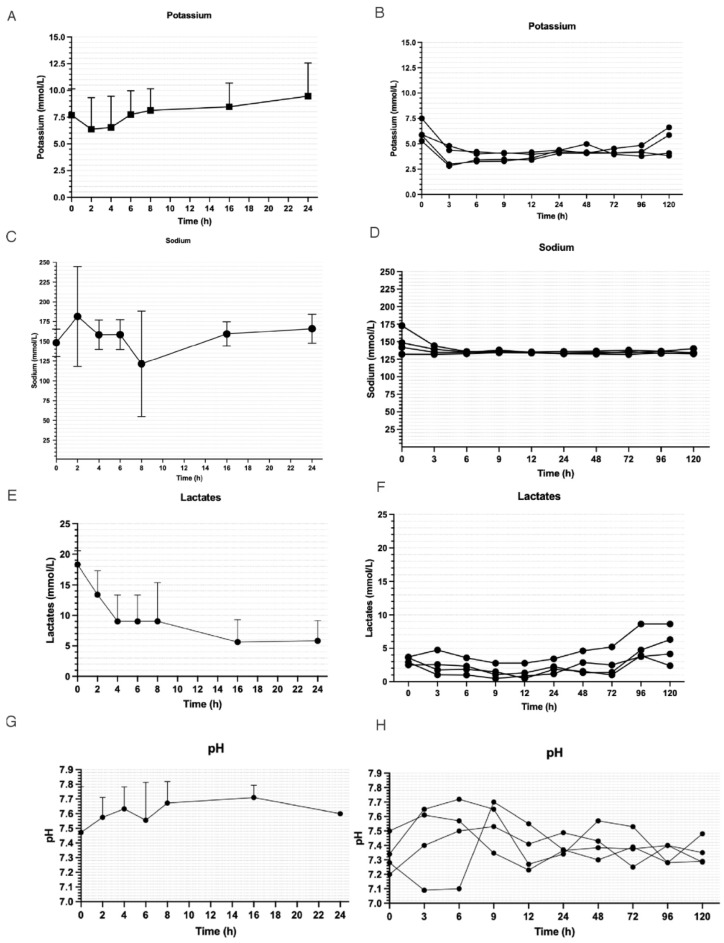
Clinical measurements of potassium (**A**,**B**), sodium (**C**,**D**), lactates (**E**,**F**), and pH (**G**,**H**) during NMP procedures without CKRT (**left** panels; *n* = 4) and with CKRT (**right** panels; *n* = 4). The electrolytes were more in the physiological range during the long phase of NMP (**B**,**D**,**F**) using CKRT.

In order to more effectively show the results achieved through the integration of CKRT and NMP, we present the findings from a series of preliminary and precedent experiments wherein CKRT was combined with NMP versus NMP alone at the beginning of liver perfusion. In Figure 6, we reported the potassium, sodium, lactate levels, and pH in four liver perfusions of 24 h without CKRT and four liver perfusions with CKRT that reached 120 h without technical and biochemical complications. In the four cases without CKRT, liver perfusion was stopped at 24 h in all cases due to high potassium and lactate levels.

### 3.3. Liver Viability Tests

**Figure 7:**
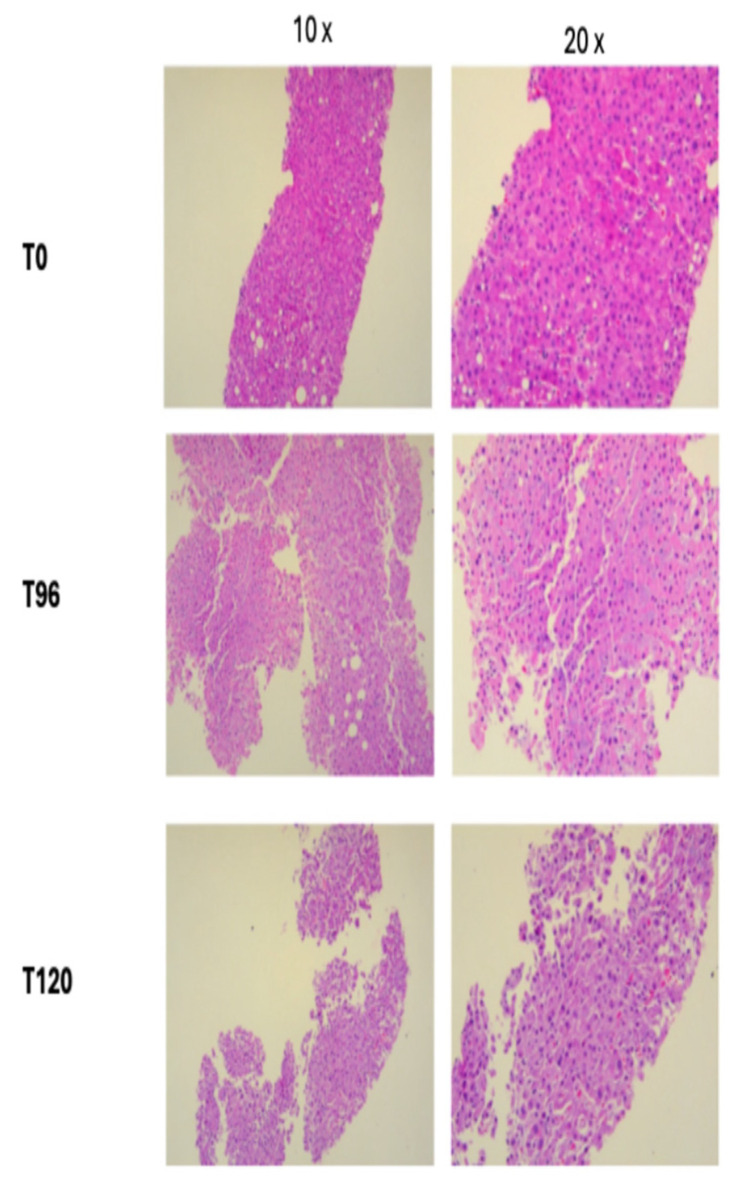
Tissue injury assessed by histological analysis (H&E) at days T0, T96, and T120.

Liver vitality was defined by the convergence of functional parameters such as good flow rates (hepatic artery and portal vein) and resistances, optimal lactate clearance, oxygen consumption, transaminases levels, hepatic synthesis factors, a good rate and quality of bile production, and the macroscopic appearance of the parenchyma. Liver biopsies (H&E) at different timepoints and at the end of perfusion demonstrated preserved architectural integrity of the liver with no evidence of substantial cell death (Figure 7). 

## 4. Discussion

We hypothesized that the NMP combined with CKRT can determine an improvement of the perfusion system reaching a physiological perfusion milieu as shown in the preliminary cases shown in Figure 6. In our study, combining normothermic liver perfusion with continuous kidney replacement therapy to maintain the best physiological perfusate for the liver perfusion determines the achievement of a physiological biochemical perfusate after the NMP priming phase and its maintenance over organ perfusion, opening the future to prolonged organ perfusion (Figure 2, Figure 3, Figure 4 and Figure 5). 

The constitution of the perfusate for NMP priming is characterized by using different solutions of fluids and drugs, resulting in the non-physiological final perfusate composition at the beginning of organ perfusion. Furthermore, the start of organ perfusion initiates the release of ions and catabolites from damaged cells, further exacerbating the perfusate’s unsafe biochemical state.

During perfusion, the metabolic changes of the organ’s cells can determine the production of numerous catabolites, which accumulate in the perfusate, causing a toxic effect on the organ’s metabolic activities. Continuously administering drugs containing sodium and sodium bicarbonate to maintain the acid–base balance over time increases perfusate osmolality, leading to variations in osmotic strength and water movement from the intracellular to the extracellular space. These changes may have repercussions on cellular metabolism and organ vitality. The continuous removal of molecules from the perfusate for physiologic and metabolic activity (glucose, vitamins, amino acids, etc.) may necessitate the continued administration of these molecules in conjunction with their frequent monitoring of levels, determining an overload of biochemical control and correction activities that have to be implemented by the technician in charge of perfusion. Furthermore, cellular energy metabolism produces a series of molecules usually eliminated by the kidney (urea, uric acid, etc.). In the absence of this artificial organ, there is an increase in these molecules in the perfusate with possible metabolism alterations as occurs in vivo during renal failure.

**Figure 8:**
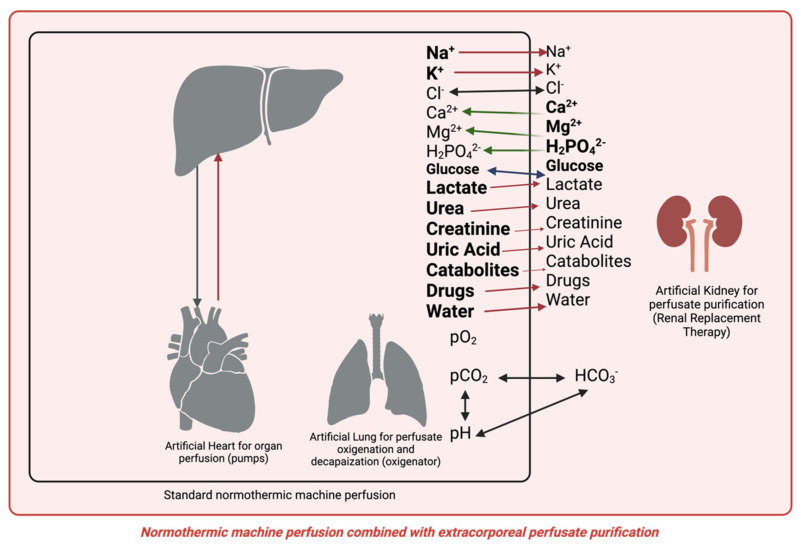
Combination of CKRT and NMP for the purification of perfusate and maintenance of hydro-electrolyte balance and acid–base status.

Current NMP technologies initiate perfusion with non-physiological perfusates (Figure 2, Figure 3, Figure 4 and Figure 5), with further biochemical deterioration occurring during perfusion, particularly in prolonged treatments or when cellular metabolism slows. The potential for integrating a sophisticated extracorporeal purification system with NMP offers a promising avenue for addressing these challenges. The CKRT provides the potential for the continuous and efficient correction of hydro-electrolyte and acid–base imbalances. At the outset of the NMP treatment, this technique can restore a physiological perfusate despite its intrinsic initial composition. Subsequently, CKRT can maintain the hydro-electrolyte and acid–base balance of the integrated organ-NMP-CKRT system at a physiological condition by the purification requirements of the organ’s metabolic processes. The potential for tailoring the CKRT in terms of purification dose, hemodiafiltration electrolytic fluid composition, fluid removal, and filter characteristics enables the creation of a versatile, integrated, and dynamic system that is capable of not only removing catabolites or ions in excess but also of providing those substances that are continuously used by cellular metabolisms, such as glucose and phosphate (Figure 8).

The utility of CKRT is of particular significance in the context of metabolic conditions where the rapid removal of organic acids or ions (such as lactic acid or potassium) is necessary, for instance, during temporary anaerobic metabolism due to functional alterations of the organ or the infusion of concentrated red blood cells to maintain the hematocrit in long-term perfusions. Of particular interest is the integration of CKRT in maintaining acid–base balance and controlling CO_2_ levels. Integrating bicarbonate control through back filtration in diffusion or reinfusion in post-dilution enables the pH to be regulated by acting not only on CO_2_ levels but also by continuously infusing bicarbonates into the system. Changing the purifying dose and the different ratios between convective and diffusive doses makes it possible to manage the gain of bicarbonates and all the ions.

From this perspective, the CKRT system can be considered a tool for maintaining the perfusion milieu of the organ ex vivo, as occurs in vivo by the lung–liver–kidney and heart interaction configuring a new generation of machines for the normothermic organ perfusion.

The principal limitation of the system pertains to its inability to reabsorb filtered beneficial substances and synthesize molecules usually produced by the kidney (erythropoietin, vitamin 1,25-OHD, etc.). Controlling the hydro-electrolyte and acid–base balance necessitates the implementation of frequent biochemical assessments and customizing the electrolyte and bicarbonate content of the fluids employed for purification, thereby increasing the complexity of the method. The loss of low-molecular-weight nutrients has to be considered and compensated by periodic or continuous infusion of such substances into the system to ensure the organ maintains active metabolism.

Despite these limitations, it is currently possible to exploit the characteristics of CKRT to obtain perfusate purification by combining CKRT with an NMP system. This allows for achieving and maintaining a physiological perfusate during prolonged perfusion, guaranteeing the best metabolic conditions for the perfused organ. It is, therefore, possible to conclude that the combination of CKRT with an NMP system represents a promising approach for perfusate purification, with the potential to achieve and maintain a physiological organ milieu during prolonged perfusion and different metabolic requirements or during organ therapy that can induce cell damage, inflammation, and cytokine production.

The available hardware allows us to obtain an organ perfusion and perfusion purification system by associating various specific modules that perform the individual organ functions (Figure 8). These include the heart for perfusion, the lungs for oxygenation and carbon dioxide removal, and the kidneys for purification. In the future, it will be necessary to miniaturize kidney purification systems by reworking the physical processes [30] underlying purification to obtain miniaturized devices [31,32] that can be integrated into perfusion modules to make them transportable and usable in all clinical settings, ensuring dynamic purification from toxic substances also in a continuous or prolonged time of application [33,34,35].

The CKRT-NMP integrated system presented here has the invaluable potential to be transformative as a platform for regenerative medicine approaches, enabling repair and reconditioning of injured donor livers, immunomodulation, gene product delivery, and other treatments in the future. The quality of the homeostasis obtained throughout the experiment is promising and paves the way for future ex vivo organ manipulation interventions. Furthermore, it is worth noting that all the integrated elements, including the perfusion platform (Liver Assist, Xvivo), the oxygenator, and the blood purification system, are approved for clinical use and commercially available, resulting in a potentially easier translation to a clinical phase.

The present study demonstrates the feasibility of the first step in maintaining organ perfusion for extended periods by preserving a physiological perfusate over time. We think that the use of customized perfusate purification technology (CKRT), based on diffusion and convection with post-dilution reinfusion, can allow a physiological perfusate to be obtained and maintained according to the metabolic needs of the perfused organ. Future studies will have to evaluate the impact of this technology on organ function, which may be based on integrated CKRT-NMP systems that make the perfusion system more physiological than those currently available. 

The present study confirms, as a first step, the technical feasibility of sustaining a physiological perfusate for extended perfusion periods. The clinical implications and efficacy of this technique must be further investigated in a setting where NMP-treated organs are utilized for transplantation. At present, the organs perfused within the scope of this project are not allocated for transplantation. 

## 5. Conclusions

Combining CKRT with NMP offers a promising approach for perfusate purification, achieving and maintaining a physiological organ milieu during perfusion. This system ensures optimal metabolic conditions for the perfused organ, addressing the varying metabolic requirements and potential cell damage, inflammation, and cytokine production. The current hardware facilitates an integrated organ perfusion and purification system, mimicking the functions of the heart, lungs, and kidneys.

Future developments should focus on miniaturizing kidney purification systems to enhance portability and clinical applicability. The CKRT-NMP integrated system holds transformative potential for regenerative medicine, enabling the repair and reconditioning of injured donor livers, immunomodulation, gene product delivery, and other advanced treatments. The successful homeostasis observed throughout our experiment suggests a promising future for ex vivo organ manipulation and clinical translation supported by commercially available and clinically approved components.

## Data Availability

The data that support the findings of the study are available from the corresponding author upon reasonable request.
